# When Pneumonia Isn’t the Whole Story: A Diagnostic Challenge in Septic Shock

**DOI:** 10.7759/cureus.94999

**Published:** 2025-10-20

**Authors:** Pedro Pires Mesquita, Gonçalo Torrinha, Vânia Gomes

**Affiliations:** 1 Internal Medicine, Unidade Local de Saúde de Braga, Braga, PRT; 2 Intensive Care, Unidade Local de Saúde de Braga, Braga, PRT

**Keywords:** bloody diarrhea, diagnostic and therapeutic challenge, elderly patient care, emergency gastroenterology and endoscopy, foreign body retrieval, immunosuppression, septic shock [ss], severe community-acquired pneumonia

## Abstract

Septic shock in immunocompromised patients typically arises from common infections but may coexist with unexpected findings that influence clinical reasoning and management. We report the case of a 77-year-old male patient with rheumatoid arthritis under chronic immunosuppressive therapy (low-dose corticosteroids and methotrexate), admitted to the intensive care unit with septic shock and multiorgan dysfunction. The clinical presentation at the emergency department, with two days of fever and productive cough, respiratory failure, and hypotension, was consistent with severe community-acquired pneumonia. This was confirmed by imaging, which showed right-lower-lobe pulmonary infiltrates and pleural effusion.

However, the patient also described a seven-day history of diarrhea with mucus and blood, prompting further gastrointestinal investigation. Abdominopelvic CT revealed sigmoid diverticulosis and a thin, linear hyperdense structure embedded in the colonic wall. Flexible sigmoidoscopy confirmed the presence of a filiform foreign body, compatible with a fruit stem, approximately 3 cm in length, which was successfully removed.

Although pneumonia was considered the primary source of septic shock, the gastrointestinal findings may have contributed to systemic inflammation in a vulnerable host. This case reinforces the need for a broad diagnostic evaluation in septic patients with overlapping symptoms and highlights the relevance of incidental findings in complex clinical contexts.

## Introduction

Not every case unfolds the way we expect. In older or immunosuppressed patients, even something as common as pneumonia can come with twists that challenge the clinical narrative. What initially appears straightforward can sometimes reveal additional layers that demand closer attention.

Foreign body ingestion is something we are used to seeing in children, but it is far from rare in adults, particularly in those who are elderly, cognitively frail, or have underlying neurological or psychiatric conditions. In most situations, the object passes without incident. Yet, in a small number of cases, these ingestions can lead to local irritation, inflammation, or even more serious outcomes like infection, perforation, or systemic illness. In people with compromised immune systems, even minor mucosal injuries can tip the balance, allowing bacterial translocation and systemic inflammation to take hold [1,2].

While pneumonia, urinary tract infections, and abdominal sepsis remain the usual suspects in cases of septic shock, clinicians should remain open to alternative or coexisting sources, especially when symptoms span more than one system. In patients with vague or overlapping complaints, particularly those on long-term immunosuppression, we need to think broadly and avoid prematurely anchoring to the obvious diagnosis.

There are examples in literature that illustrate this risk. One patient developed a liver abscess after unknowingly ingesting a fish bone that had perforated the stomach wall [1]. Another immunosuppressed adult was found to have a biliary stent lodged in the sigmoid colon, creating a local inflammatory response [2]. In other reports, elderly patients developed pneumonia and septic shock following the aspiration of plant material, with resolution only after bronchoscopic removal [3,4].

In this report, we describe a 77-year-old male patient with rheumatoid arthritis, on chronic corticosteroids and methotrexate, who was admitted with septic shock from community-acquired pneumonia. But his case didn’t stop there. Persistent gastrointestinal symptoms led to an unexpected finding: a small, linear foreign body lodged in the wall of the sigmoid colon. Although pneumonia was clearly the main driver of his condition, this gastrointestinal finding was far from irrelevant. In a patient with impaired immune defenses, even a minor mucosal injury can add fuel to the inflammatory fire. This case reminds us to stay curious and thorough, because sometimes, a secondary detail ends up adding meaningful context to the whole picture.

## Case presentation

A 77-year-old male patient with a history of chronic obstructive pulmonary disease and rheumatoid arthritis, medicated with 5 mg of prednisolone daily and 10 mg of methotrexate weekly, presented to the emergency department with diarrhea lasting approximately seven days. He reported four to five bowel movements per day and had noticed blood on the toilet paper the day before. Concurrently, he developed a two-day history of cough with yellow sputum, shortness of breath, and an isolated febrile episode.

On arrival at the emergency department, he was drowsy but responsive to verbal stimuli. He was pale, diaphoretic, and had mottled skin. Vital signs were as follows: blood pressure 71/47 mmHg (reference range: 90-120/60-80 mmHg), heart rate 84 bpm (60-100 bpm), tachypnea requiring high-flow oxygen mask at 15 L/min with peripheral oxygen saturation of 92%. Due to respiratory fatigue, non-invasive ventilation (Inspiratory Positive Airway Pressure/Expiratory Positive Airway Pressure (IPAP/EPAP) of 12/8, Fraction of Inspired Oxygen (FiO₂) of 60%) was initiated. Pulmonary auscultation revealed rhonchi, predominantly in the right lung. Abdominal palpation revealed generalized tenderness without signs of peritoneal irritation. Fluid resuscitation was initiated.

His laboratory findings are presented in Table 1. 

**Table 1:** Results of the laboratory values AST: Aspartate aminotransferase; GOT: Glutamic oxaloacetic transaminase; ALT: Alanine aminotransferase; GPT: Glutamic pyruvic transaminase; LDH: Lactate dehydrogenase; CK: Creatine kinase; NT-proBNP: N-terminal pro-B-type natriuretic peptide.

Parameter	Maximum observed value	Reference range
Hemoglobin (g/dL)	11.7	13.5-17.0
Leukocytes (×10³/μL)	14.7	4.0-11.0
Neutrophils (%)	88	1.8-7.1
Platelets (×10³/μL)	461	150-400
C-reactive protein (mg/L)	162	<5.0
Procalcitonin (ng/mL)	50.36	<0.05
Creatinine (mg/dL)	1.8	0.7-1.2
Urea (mg/dL)	89	19-49
Glucose (mg/dL)	224	82-115
Sodium (mmol/L)	139	136-145
Potassium (mmol/L)	4.6	3.5-5.1
Chloride (mmol/L)	108	98-107
AST (GOT) (U/L)	27	12-40
ALT (GPT) (U/L)	21	7-40
Amylase (U/L)	173	30-118
Lipase (U/L)	24	12-53
LDH (U/L)	284	120-246
Total CK (U/L)	31	46-171
Troponin I (ng/mL)	0.235	<0.045
Myoglobin (ng/mL)	296	<110
NT-proBNP (pg/mL)	1611	<450

**Figure 1:**
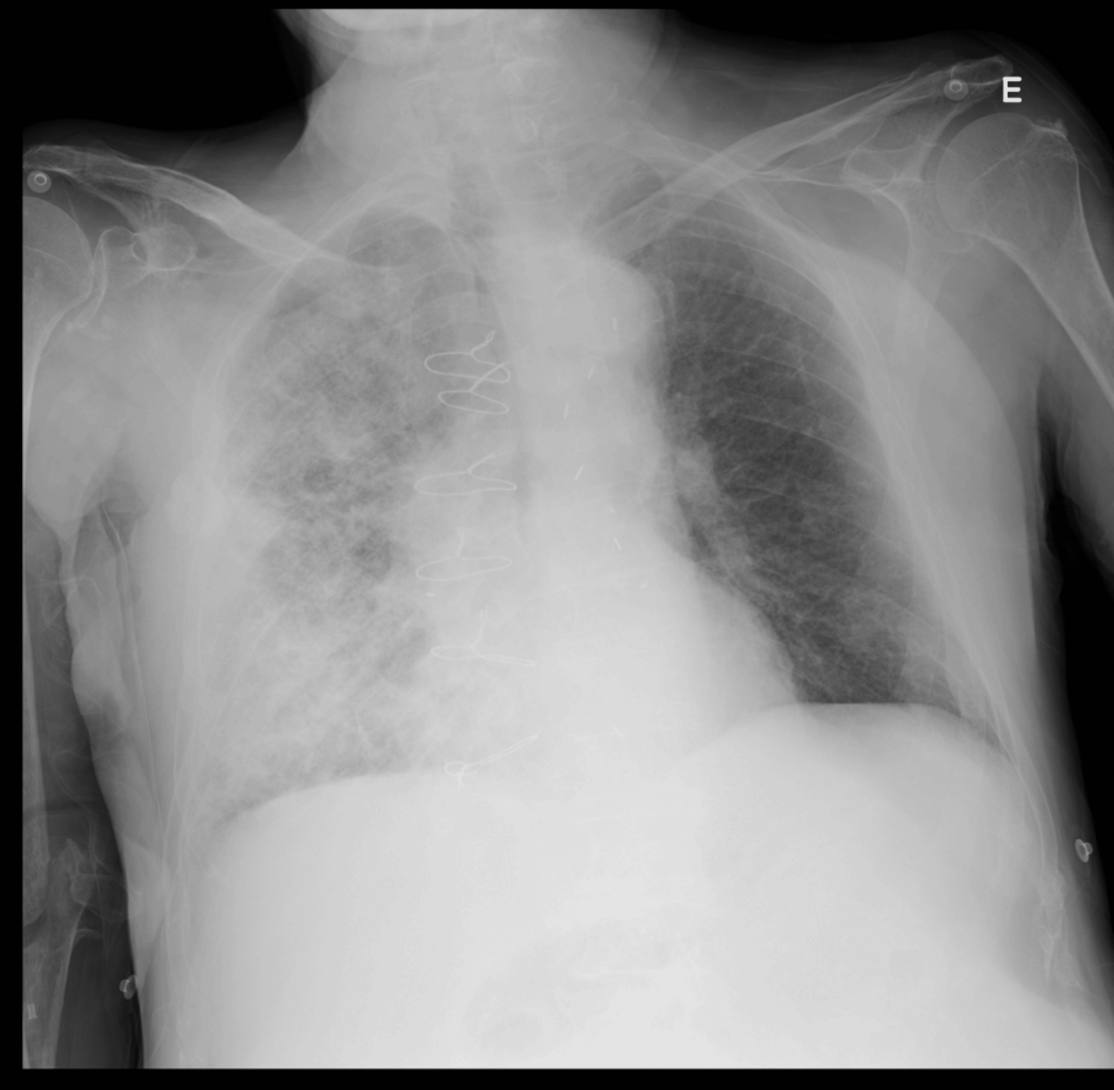
Chest X-ray (anteroposterior view) The chest X-ray revealed widespread opacities on the right side, more prominent in the middle and lower lung zones, suggesting consolidation. There was also a moderate pleural effusion on the right, evident by the blurring of the costophrenic angle and the silhouette of the right hemidiaphragm. The left lung appeared mostly clear. The heart size was within normal limits, and there were no signs of pneumothorax.

An initial chest X-ray (Figure 1) revealed widespread opacities on the right side, more prominent in the middle and lower lung zones, suggesting consolidation.

The Intensive Care Unit (ICU) was contacted, and the patient was admitted to the emergency room for septic shock with multiorgan dysfunction (renal, neurological, cardiovascular, and respiratory) stemming from community-acquired pneumonia in an immunocompromised patient. We decided to initiate vasopressor support with norepinephrine, administered a 200 mg bolus of hydrocortisone, and started empirical antibiotic therapy with piperacillin-tazobactam and linezolid. Blood cultures, sputum microbiology, and urinary antigen tests for Streptococcus pneumoniae and Legionella were collected. The patient was transferred to the ICU (Level 3 care).

**Figure 2:**
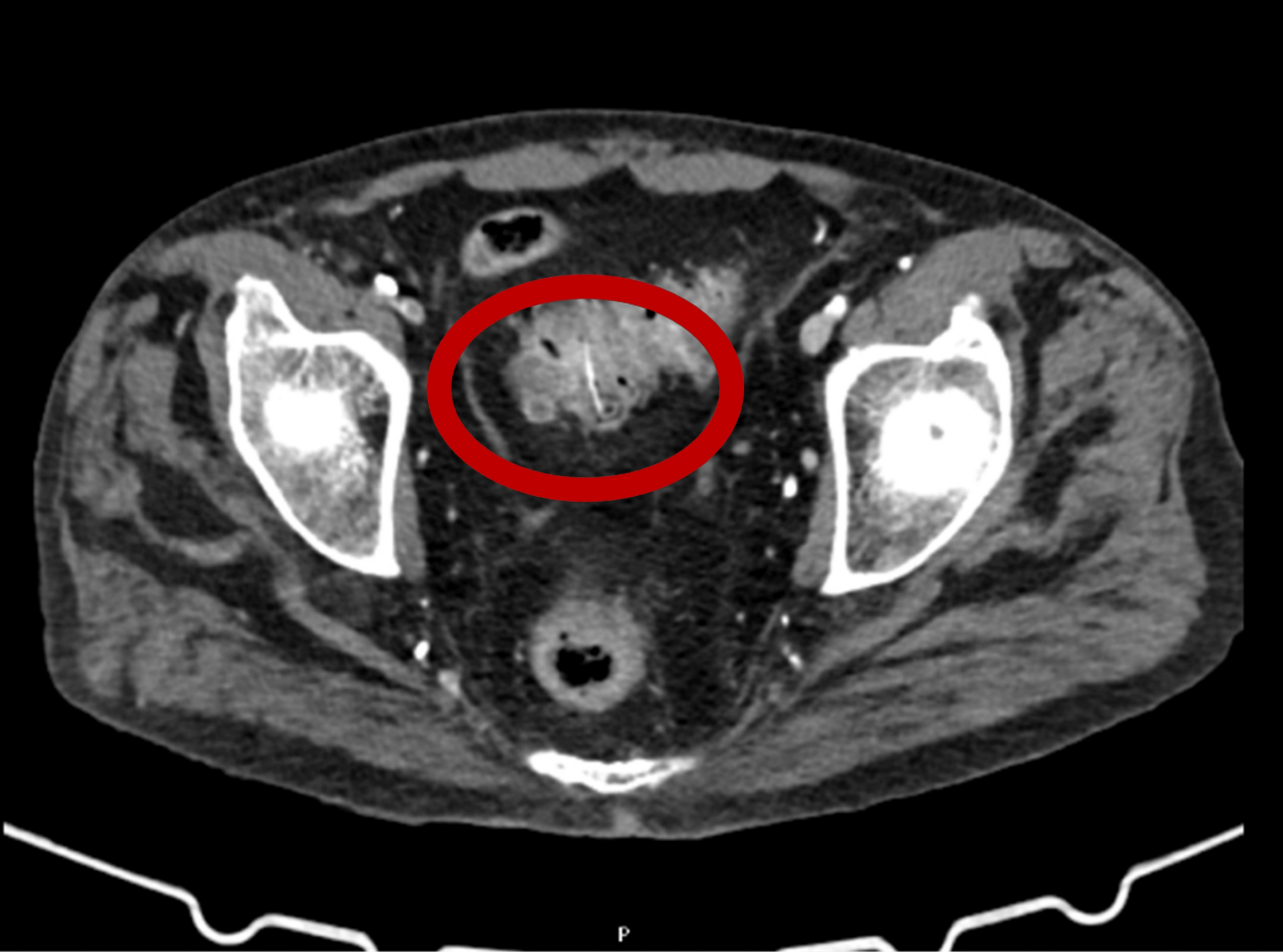
CT of the thorax, abdomen, and pelvis (CT-TAP) CT-TAP showing a faint, linear, dense image along the inferior border of the sigmoid colon wall (intraparietal) in its distal half, filiform in shape (longitudinal extension of 2.4 cm), of nonspecific nature, and not associated with the surrounding fluid collection.

Given the gastrointestinal component alongside respiratory symptoms, a thoraco-abdominopelvic CT scan was ordered. The CT scan revealed multiple irregularly contoured opacities in the right lung, predominantly at the bases, suggestive of inflammatory/infectious lesions or nonspecific pneumonitis with pleural effusion. Diverticulosis of the sigmoid colon with mild wall thickening raised suspicion of early diverticulitis. A linear, faintly dense structure, approximately 2.4 cm in length, was observed along the inferior border of the sigmoid colon wall (intraparietal), with no associated fluid collections (Figure 2).

**Figure 3:**
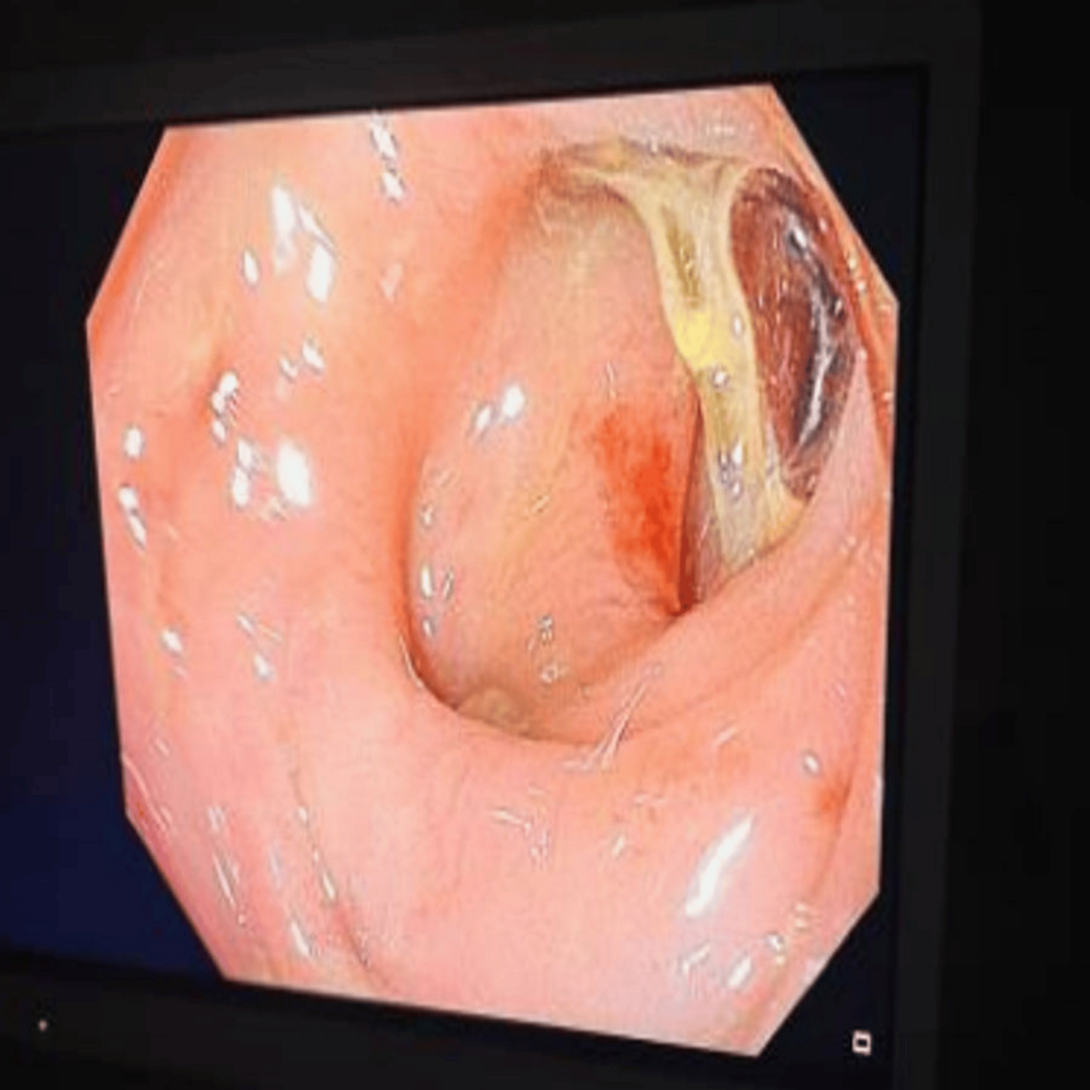
Flexible sigmoidoscopy image The endoscopic image shows a linear, filiform foreign body embedded in the wall of the sigmoid colon, approximately 20 cm from the anal verge. The object appears to have a firm and slightly curved structure, compatible with a plant stem. There is mild erythema and mucus surrounding it, but no signs of perforation, ulceration, or active bleeding. These findings are consistent with localized mucosal irritation caused by the foreign body.

**Figure 4:**
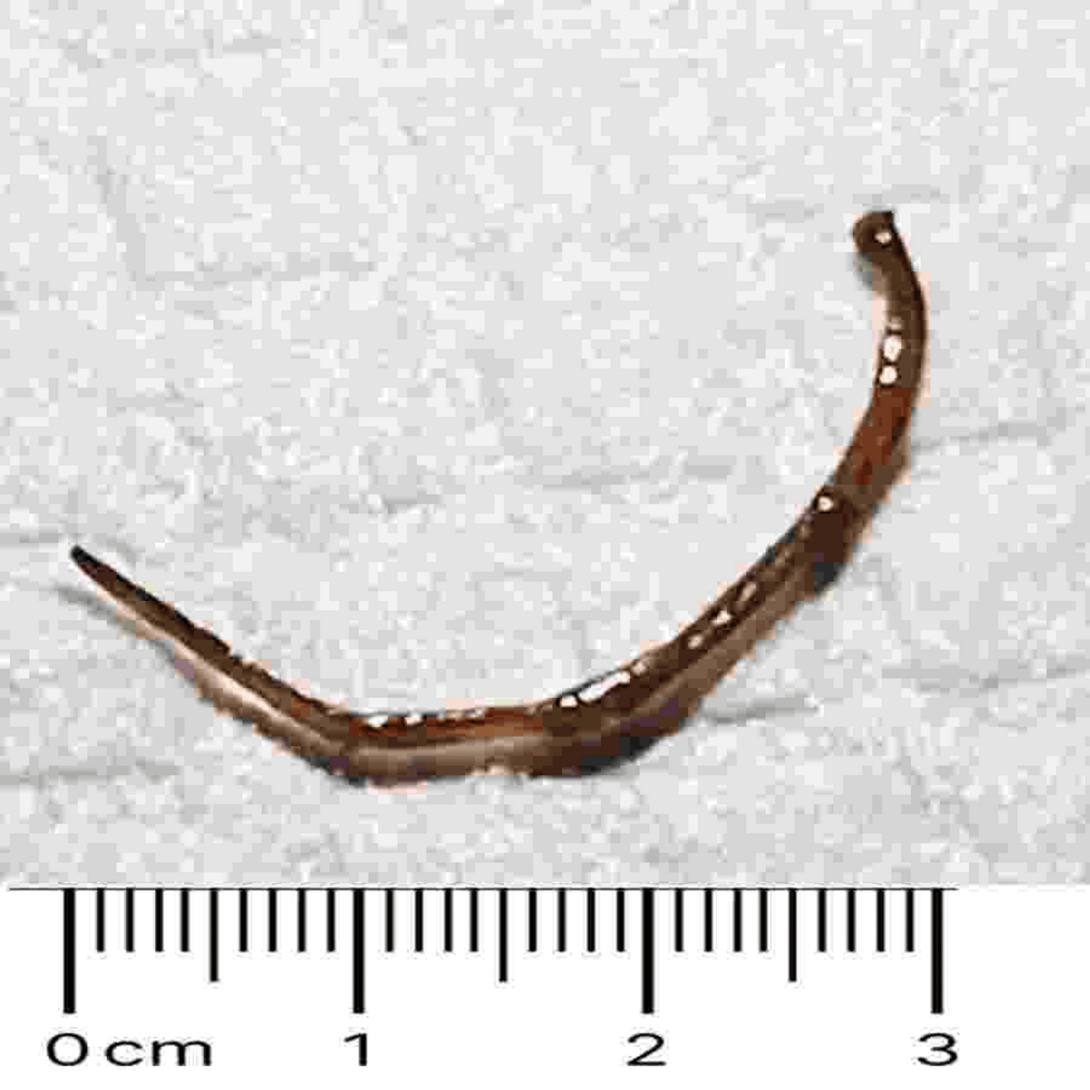
Foreign body A filiform and slightly rigid foreign body (fruit stem), approximately 3 cm in length, removed during rectosigmoidoscopy at 20 cm from the anal margin.

Gastroenterology was consulted, and the patient underwent rectosigmoidoscopy. Perianal inspection and rectal examination were unremarkable. A thin, slightly firm (approximately 3 cm, resembling a fruit stem) was identified 20 cm from the anal margin (Figure 3) and removed with forceps (Figure 4).

No signs of acute diverticulitis were noted.

The patient showed clinical improvement, with vasopressor support discontinued on day three of hospitalization. By day five, he was transferred to the Internal Medicine ward with improved renal function, oxygen saturation >94% on room air, and no further episodes of diarrhea.

## Discussion

Sepsis doesn’t always follow a predictable path. In real-world clinical settings, especially in older, frail, or immunosuppressed patients, what seems like a clear-cut infection can hide unexpected details that force us to rethink our initial assumptions. This patient presented with a typical picture of community-acquired pneumonia: fever, purulent cough, and respiratory failure. The chest X-ray confirmed right-sided consolidation, and the inflammatory markers, including a procalcitonin level above 50 ng/mL (reference value: <0.05 ng/mL), pointed to a severe bacterial infection. The diagnosis of septic shock was based on clinical criteria consistent with sepsis-3 definitions: suspected infection, hypotension requiring vasopressor support, and elevated lactate in the setting of multiorgan dysfunction [5].

But alongside these respiratory symptoms, the patient also described seven days of diarrhea with mucus and blood. Rather than chalk this up to a benign issue, we investigated further. Abdominal imaging revealed a thin, linear, hyperdense structure embedded in the wall of the sigmoid colon. On endoscopy, we found what looked like a plant stem, filiform, about 3 cm long, lodged 20 cm from the anal verge. In most patients, this might have been an incidental finding. However, in someone on chronic corticosteroids and methotrexate, even a small mucosal injury can become a gateway for bacterial translocation and systemic inflammation [6,7].

One of the things we had to consider was whether there could be more going on than just pneumonia. The chest X-ray showed a dense, right-sided consolidation, clearly indicating a severe infection. But the patient’s oxygenation was disproportionately poor, with a PaO₂/FiO₂ ratio of 144 despite high-flow oxygen through non-invasive ventilation. In a setting of sepsis and such profound hypoxemia, we couldn't ignore the possibility of evolving or overlapping Acute Respiratory Distress Syndrome (ARDS).

ARDS can arise in the setting of sepsis through a cascade of inflammatory responses. Cytokines like tumor necrosis factor (TNF)-α, IL-1β, and IL-6 are released into circulation, activating the pulmonary endothelium, increasing capillary permeability, and attracting neutrophils into the lungs. These neutrophils release proteases and reactive oxygen species, damaging the alveolar-capillary membrane. The result is non-cardiogenic pulmonary edema, impaired oxygenation, and in severe cases, respiratory failure [8]. In immunocompromised patients, this process can unfold with fewer radiological findings, even as hypoxemia becomes life-threatening.

In our case, it’s possible that the gastrointestinal inflammation, caused by the foreign body, acted as a secondary inflammatory trigger, further fueling the systemic response. This concept of a “double-hit” mechanism, where simultaneous pulmonary and extrapulmonary inflammation worsen alveolar injury, has been described in the context of sepsis-related ARDS [8].

There are similar cases in the literature. One describes a liver abscess caused by a swallowed fish bone that migrated through the stomach wall [6]. Another details an immunosuppressed adult with a biliary stent found in the sigmoid colon, causing inflammation without perforation [7]. Even in the respiratory tract, foreign body aspiration in elderly patients has led to pneumonia and septic shock, only resolving after removal [3,4]. These cases highlight how something as small and easily overlooked as a swallowed object can lead to major complications.

Although pneumonia was clearly the main source of this patient’s sepsis, the gastrointestinal finding wasn’t irrelevant. It may not have caused the sepsis alone, but it likely contributed to the overall inflammatory burden or at the very least, added complexity to an already fragile situation. This case serves as a reminder that when patients present with symptoms across multiple systems, especially when they’re immunocompromised, we need to look beyond the obvious and remain open to alternative or additional diagnoses.

The patient's recovery, following broad-spectrum antibiotics, vasopressor support, and endoscopic removal of the foreign body, reinforces the value of that kind of clinical thinking. Sometimes, what seems like a secondary detail turns out to be part of the bigger picture and worth acting on.

## Conclusions

This case reminded us that even when a diagnosis seems clear, there may still be more to uncover. The patient had every sign of severe pneumonia, and that alone could explain his septic shock. But the presence of persistent gastrointestinal symptoms led to the unexpected finding of a foreign body in the sigmoid colon, something we could have easily missed, but chose to explore.

In someone immunosuppressed and already struggling with a systemic infection, even a small mucosal injury might have added to the inflammatory burden. And while the radiograph pointed strongly to lobar pneumonia, the depth of his hypoxemia pushed us to consider whether ARDS might also be evolving. The two conditions aren’t mutually exclusive; they can happen side by side and often do in septic patients.

What stayed with us from this case is how important it is to keep an open mind. Sometimes the details that seem secondary are part of the bigger picture. And in patients with multiple systems affected, especially those who are vulnerable, it’s often the extra step, the second question, or the decision to look twice that makes the difference.
